# Machine learning-based prediction of mortality risk in AIDS patients with comorbid common AIDS-related diseases or symptoms

**DOI:** 10.3389/fpubh.2025.1544351

**Published:** 2025-03-12

**Authors:** Yiwei Chen, Kejun Pan, Xiaobo Lu, Erxiding Maimaiti, Maimaitiaili Wubuli

**Affiliations:** ^1^Department of Epidemiology and Health Statistics, School of Public Health, Xinjiang Medical University, Urumqi, Xinjiang, China; ^2^Department of Infectious Diseases, The First Affiliated Hospital of Xinjiang Medical University, Urumqi, Xinjiang, China

**Keywords:** machine learning, XGBoost, AIDS, HIV, prediction model

## Abstract

**Objective:**

Early assessment and intervention of Acquired Immune Deficiency Syndrome (AIDS) patients at high risk of mortality is critical. This study aims to develop an optimally performing mortality risk prediction model for AIDS patients with comorbid AIDS-related diseases or symptoms to facilitate early intervention.

**Methods:**

The study included 478 first-time hospital-admitted AIDS patients with related diseases or symptoms. Eight predictors were screened using lasso regression, followed by building eight models and using SHAP values (Shapley’s additive explanatory values) to identify key features in the best models. The accuracy and discriminatory power of model predictions were assessed using variable importance plots, receiver operating characteristic curves, calibration curves, and confusion matrices. Clinical benefits were evaluated through decision-curve analyses, and validation was performed with an external set of 48 patients.

**Results:**

Lasso regression identified eight predictors, including hemoglobin, infection pathway, Sulfamethoxazole-Trimethoprim, expectoration, headache, persistent diarrhea, Pneumocystis jirovecii pneumonia, and bacterial pneumonia. The optimal model, XGBoost, yielded an Area Under Curve (AUC) of 0.832, a sensitivity of 0.703, and a specificity of 0.799 in the training set. In the test set, the AUC was 0.729, the sensitivity was 0.717, and the specificity was 0.636. In the external validation set, the AUC was 0.873, the sensitivity was 0.852, and the specificity was 0.762. Furthermore, the calibration curves showed a high degree of fit, and the DCA curves demonstrated the overall high clinical utility of the model.

**Conclusion:**

In this study, an XGBoost-based mortality risk prediction model is proposed, which can effectively predict the mortality risk of patients with co-morbid AIDS-related diseases or symptomatic AIDS, providing a new reference for clinical decision-making.

## Introduction

The risk of death from AIDS has been a significant global concern for decades. Among these, AIDS-related diseases and symptoms are important factors that affect the prognosis of patients. The National Institutes of Health (NIH) invested nearly $69 billion in AIDS research between 1982 and 2018 to understand, treat, and prevent HIV infection (1, 2). Studies have demonstrated that HIV infection progressively compromises the patient’s immune system, with a gradual depletion of CD4+ T-lymphocytes (3–5). This ultimately leads to various opportunistic infections (OIs) and tumors. Since 1980, OIs have accounted for a large proportion of deaths among HIV-infected patients, particularly in Asia and sub-Saharan Africa and West Africa (6–9).

OIs encompass a variety of bacteria, viruses, fungi, and parasites, some of which are exceedingly rare in immunocompetent populations (10). OIs pose a significant health risk to patients with AIDS, particularly when the CD4+ T-cell count is below 200 cells/μL. The presence of various OIs significantly increases mortality rates in patients.

Tumors in people with AIDS include both AIDS-defining and non-AIDS-defining tumors, with generally low survival rates for these patients. AIDS-defining tumors mainly include Kaposi’s sarcoma and non-Hodgkin’s lymphoma. Non-AIDS-defining tumors include lung cancer, hepatocellular carcinoma, and perianal tumors, among others. AIDS-defining tumors account for 15–19% of deaths in HIV-infected patients, with most having an early onset and a more aggressive course than non-AIDS-defining tumors (11). Confirmation of AIDS-associated cancers primarily relies on histopathological biopsy (12).

With the widespread use of Combination Antiretroviral Treatment (cART), AIDS is gradually becoming a chronic disease with a limited impact on life expectancy, but this increased survival has also led to a surge in comorbidities (13–16). Early cART effectively prevents OIs and tumors, reducing the risk of developing these conditions. Consequently, the proportion of OIs and tumors in treated patients has greatly reduced, although it remains high (17–20). OIs and tumors remain the leading cause of death among people living with HIV, with significantly higher mortality rates, particularly in low- and middle-income countries (21).

Although some studies have explored factors affecting the prognosis of patients with AIDS, including hemoglobin, viral load, and CD4+ T-cell counts, they have been Cox regression and Logistic regression, which are traditional regression methods that, while providing a basic predictive framework, are usually unable to deal effectively with high-dimensional data or complex variable interactions, and especially exhibit significant limitations when nonlinear effects are involved, may not be able to effectively capture the complex relationships between variables, resulting in inadequate predictive performance (22–27). In addition, machine learning techniques have been gradually introduced into the medical field in recent years, and their advantages in large-scale data analysis and complex model construction have been widely recognized. Recently, machine learning algorithms have become increasingly popular in healthcare, with clinically based machine learning models being used for prognostic predictions in various diseases, such as diabetes mellitus and rectal cancer (28–36). The application of death prediction in infectious diseases is also becoming a growing trend, particularly for predicting patient mortality risk, as evidenced by the COVID-19 outbreak (36, 37). However, predictive studies of the risk of death for first-time HIV admissions are scarce, and no studies have used machine learning methods to predict the risk of death for first-time admissions with co-morbid HIV-related illnesses or symptoms. To fill this research gap, this study develops a machine learning-based optimal mortality risk prediction model based on a comprehensive dataset covering demographic information, clinical manifestations, and laboratory metrics, and combines it with the SHAP tool for model interpretability analysis to help clinicians identify high-risk patients and adjust their treatment plans. In addition, we validate the performance of the model with an external dataset to demonstrate its stability and generalization ability in real-world applications. XGBoost performs better in dealing with complex data structures and nonlinear relationships than other machine learning methods by integrating multiple decision trees, has higher prediction accuracy, more efficient big data processing capability, supports parallelized training, faster training speed, less resource consumption, and has strong generalization ability, therefore, this study considers constructing a prediction model based on XGBoost.

In summary, the main goal of this study is to construct a set of scientifically valid mortality risk prediction models to support clinicians in early diagnosis and individualized interventions for first-time HIV admissions to improve patient prognosis.

## Methods

### Research design

The data used to construct and test the model in this study were obtained from 478 patients with AIDS who attended the Infectious Diseases-Hepatology Center of the First Affiliated Hospital of Xinjiang Medical University between October 2000 and January 2021, presenting AIDS-related diseases or symptoms at the beginning of their admission to the hospital. We collected demographic data (e.g., sex and age), AIDS-related disease information (e.g., thrush and cryptococcosis), clinical manifestations (e.g., persistent fever and persistent diarrhea), and laboratory test data (e.g., hemoglobin and albumin) from patients at the beginning of the admission period, for a total of 55 variables. Patients were followed up regularly according to their condition after receiving cART until March 7, 2024, with death as the outcome indicator, resulted in a total of 248 survivors and 230 deaths. Inclusion criteria were as follows: (1) patients had a positive HIV antibody confirmatory test; (2) patients’ diagnoses of relevant opportunistic infections and tumors were based on clinical manifestations, ancillary investigations, and medical records, confirmed by discharge diagnosis; (3) patients had completed relevant investigations before receiving cART; and (4) patients had good adherence to the study and received timely follow-up visits. Patient treatment adherence was measured by patients’ medication use records and regular follow-up data, which were conducted every 3 months. Exclusion criteria were as follows: (1) seriously missing case information; (2) patients with poor adherence.

### Statistical methods

Continuous variables in this study were expressed as mean ± SD or median (interquartile range), and categorical variables as frequency (percentage). These variables were then compared between survivors and deceased using Student’s t-tests, Mann–Whitney U-tests, chi-square tests, and Fisher’s exact tests. All analyses other than comparisons between multiple models were conducted in R 4.3.1, with the CBCgrps package (version 2.8.2) used for the analysis of differences (38, 39). Comparisons between multiple models were performed in Extreme Smart Analysis. All tests were two-sided with a significance level of *α* = 0.05.

### Data preprocessing

Excluded variables with more than 20% of the original data missing. The remaining data were filled using the Random Forest method. Then, the training and test sets were split in a ratio of 6:4. The Random Forest approach can effectively handle missing values through multiple interpolation and can maintain the structural integrity of the dataset. In the process of constructing each tree, Random Forest considers different feature subsets to reduce the impact of outliers, while missing values can be handled by the combined results of multiple trees.

### Selection of predictors

Using whether death was the dependent variable, first, Receiver Operating Characteristic (ROC) curves and Area Under Curve (AUC) values for all covariates in the complete dataset were generated to gain preliminary insights into the variables. Then, predictors were identified from the variables in the training set using Lasso regression and a min-max normalization was applied to the quantitative data. According to the 10 Events Per Variable (10EPV) rule, the sample size of deceased patients in the training set was ensured to meet the criterion of 10 times the number of predictors.

### Modeling

In this study, seven machine learning algorithms (XGBoost, LightGBM, AdaBoost, MLP, SVM, GNB, and KNN) and one traditional regression algorithm (Logistic Regression) were used to initially construct patient mortality risk prediction models. The optimal model was selected through 10-fold Cross-Validation (CV), and then the optimal model was subjected to hyperparameter optimization (including max_depth, eta, gamma, colsample_bytree, min_child_weight, and subsample parameters) to construct the final model. The model was interpreted using the SHAP tool. Finally, a variable importance plot, ranking graph, and variable dependency plot were generated to show the relative importance of each feature in the model.

### Evaluation of the model

The AUC is calculated from the ROC curve. The ROC curve is frequently used to assess the discriminative capacity of a predictive model, i.e., its ability to discriminate between different categories.

In this study, calibration curves were used to assess model fit, in which the Brier score was used as an evaluation metric; the lower the Brier score, the better the model fit.

The accuracy and discriminative power of model predictions were evaluated using a confusion matrix. Specific indicators include accuracy, sensitivity, specificity, positive predictive value, negative predictive value, and F1 score.

The study used Decision Curve Analysis (DCA) to evaluate the clinical utility of the model. The DCA curve plots the threshold probability on the horizontal axis and the net benefit on the vertical axis. The closer the curve is to the upper right corner, the greater the utility of the predictive model.

### External validation

Forty-eight AIDS patients presenting with AIDS-related diseases or symptoms upon their first admission to the Shayibak District Branch of Urumqi Friendship Hospital (21 survivors and 27 deceased) were included in the external validation cohort. The external validation cohort came from different hospitals in the same area and had similar disease characteristics as the training set. We incorporated the data from the external validation set into the model constructed from the training set, and assessed the performance, goodness of fit, and clinical benefit of the model by plotting ROC curves, calibration curves, and DCA curves; a higher AUC value and a high degree of fit, and a wide interval of the DCA curves illustrated a high degree of generalizability of the model.

### Ethics statement

The study protocol was approved by the Ethical Review Committee of the First Affiliated Hospital of Xinjiang Medical University (Ethical approval number: K202409-31). All experiments were conducted in accordance with relevant designated guidelines and regulations. Due to the retrospective nature of the study, the ethical review committee of the First Affiliated Hospital of Xinjiang Medical University waived the need of obtaining informed consent.

## Results

### Research flowchart

**Figure 1:**
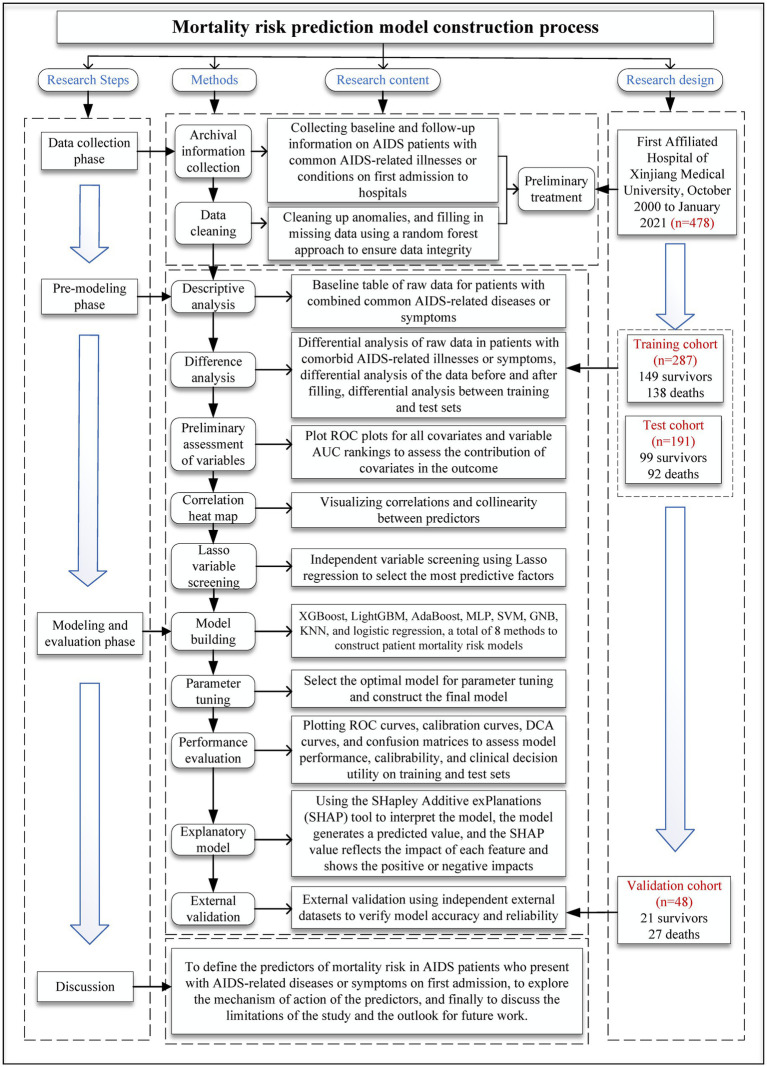
Flowchart of the mortality risk prediction model development.

The flowchart of the study is presented in Figure 1. The flowchart is divided into four sections: research steps, methods, research content, and research design.

### Patient characteristics

**Figure 2:**
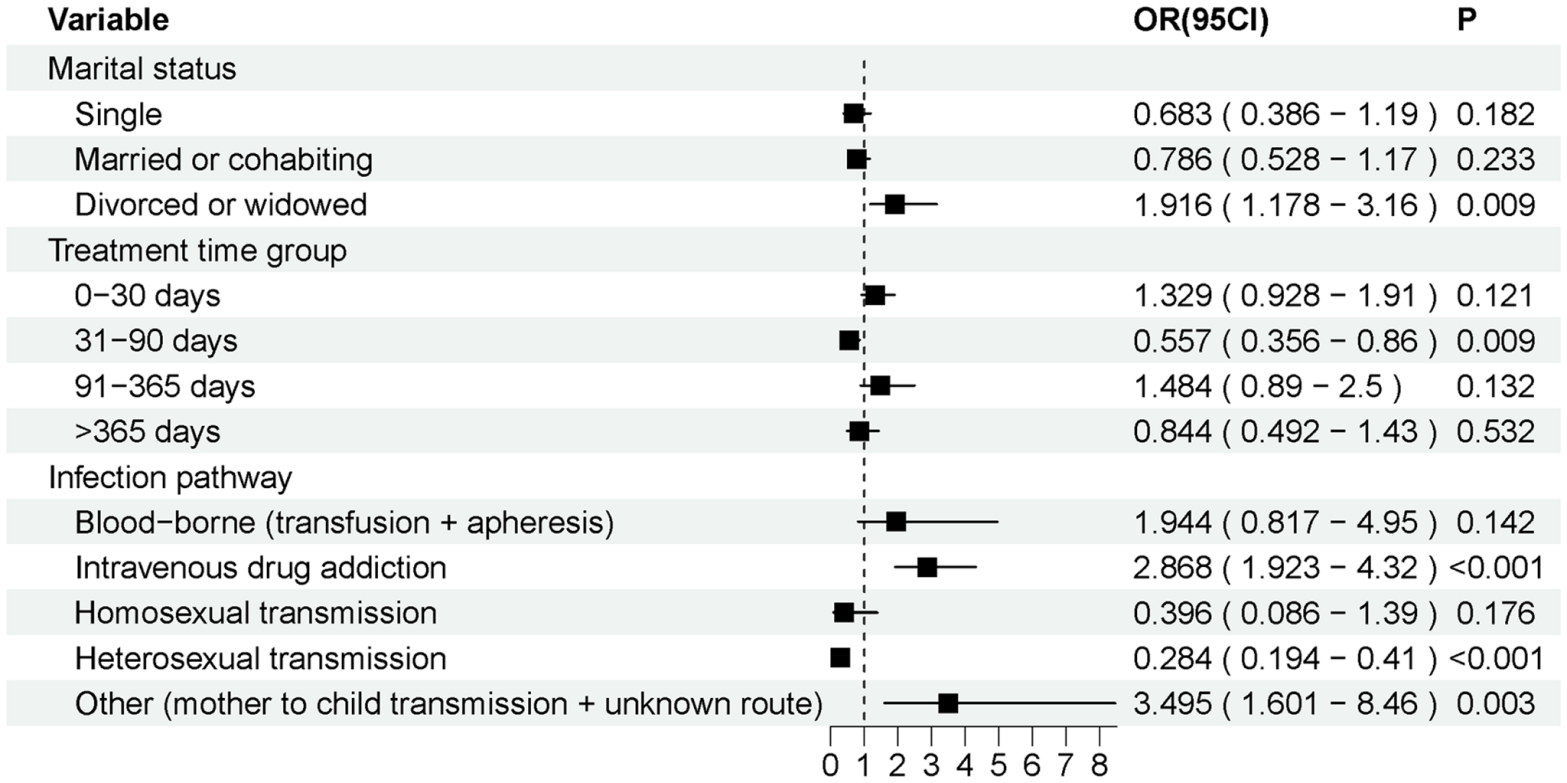
Subgroup analysis forest map. Subgroup analyses showed a significantly higher risk of death in patients who were divorced or widowed (*p* < 0.05); in patients with a time from detection of HIV positivity to initiation of treatment of 31–90 days (*p* < 0.05); and in patients whose infection pathway was intravenous drug addiction or heterosexual transmission (*p* < 0.05).

A total of 478 AIDS patients (248 survivors and 230 deceased) were included in this study up to 7 March 2024, who presented with AIDS-related diseases or symptoms at the beginning of their admission to the hospital between October 2000 and January 2021 at the Infectious Diseases-Hepatology Center of the First Affiliated Hospital of Xinjiang Medical University. The results of the analysis of variance between the two groups of raw data (Table 1) indicate that the variables of patients’ marital status, infection pathway, treatment time group, OHL, esophageal candidiasis, PJP, CMV, bacterial pneumonia, persistent diarrhea, nausea, headache, WHO stage, SMZ-TMP, and treatment plan exhibited a differential distribution between the two groups. Furthermore, deceased patients exhibited lower levels of HDL and higher levels of AST, ALT, and GGT compared to surviving patients. Subgroup analyses (Figure 2) were performed to explore variations in mortality risk across different patient characteristics. Multivariate logistic regression models were applied to estimate odds ratios (ORs) and 95% confidence intervals (CIs) for each subgroup. Variables included marital status (single, married or cohabiting, divorced or widowed), Treatment time group (0–30 days, 31–90 days, 91–365 days, >365 days), and infection pathways (blood-borne, intravenous drug addiction, homosexual transmission, heterosexual transmission, other).

**Table 1:** Baseline characterization of raw data and analysis of differences.

Variables	Total (*n* = 478)	Survival (*n* = 248)	Deceased (*n* = 230)	*p*
Marital status, n (%)				0.021
Single	58 (12.2)	35 (14.1)	23 (10.1)	
Married or cohabiting	338 (71.0)	182 (73.4)	156 (68.4)	
Divorced or widowed	80 (16.8)	31 (12.5)	49 (21.5)	
Treatment time group, n (%)				0.032
0–30 days	238 (49.8)	115 (46.4)	123 (53.5)	
31–90 days	108 (22.6)	68 (27.4)	40 (17.4)	
91–365 days	69 (14.4)	30 (12.1)	39 (17.0)	
>365 days	63 (13.2)	35 (14.1)	28 (12.2)	
Infection pathway, n (%)				<0.001
Blood-borne (transfusion + apheresis)	22 (4.6)	8 (3.2)	14 (6.1)	
Intravenous drug addiction	149 (31.2)	51 (20.6)	98 (42.6)	
homosexual transmission	11 (2.3)	8 (3.2)	3 (1.3)	
Heterosexual transmission	264 (55.2)	173 (69.8)	91 (39.6)	
Other (mother-to-child transmission + unknown route)	32 (6.7)	8 (3.2)	24 (10.4)	
OHL, n (%)				0.035
No	465 (97.3)	237 (95.6)	228 (99.1)	
Yes	13 (2.7)	11 (4.4)	2 (0.9)	
PJP, n (%)				<0.001
No	430 (90.0)	206 (83.1)	224 (97.4)	
Yes	48 (10.0)	42 (16.9)	6 (2.6)	
CMV, n (%)				0.031
No	472 (98.7)	242 (97.6)	230 (100.0)	
Yes	6 (1.3)	6 (2.4)	0 (0)	
Bacterial pneumonia, n (%)				<0.001
No	440 (92.1)	244 (98.4)	196 (85.2)	
Yes	38 (7.9)	4 (1.6)	34 (14.8)	
Persistent diarrhea, n (%)				0.017
No	418 (87.4)	226 (91.1)	192 (83.5)	
Yes	60 (12.6)	22 (8.9)	38 (16.5)	
Nausea, n (%)				0.048
No	432 (90.4)	231 (93.1)	201 (87.4)	
Yes	46 (9.6)	17 (6.9)	29 (12.6)	
Projectile vomiting, n (%)				0.053
No	474 (99.2)	248 (100)	226 (98.3)	
Yes	4 (0.8)	0 (0)	4 (1.7)	
Headache, n (%)				0.035
No	440 (92.1)	235 (94.8)	205 (89.1)	
Yes	38 (7.9)	13 (5.2)	25 (10.9)	
WHO, n (%)				<0.001
Stage 1	22 (4.6)	9 (3.6)	13 (5.7)	
Stage 2	399 (83.8)	222 (89.5)	177 (77.6)	
Stage 3	31 (6.5)	5 (2.0)	26 (11.4)	
Stage 4	24 (5.0)	12 (4.8)	12 (5.3)	
SMZ-TMP, n (%)				<0.001
No	170 (35.8)	60 (24.2)	110 (48.5)	
Yes	305 (64.2)	188 (75.8)	117 (51.5)	
Plan, n (%)				0.006
AZT + 3TC + DDI	1 (0.2)	0 (0)	1 (0.4)	
AZT + 3TC + EFV	126 (26.4)	77 (31.0)	49 (21.3)	
AZT + 3TC + LVP	3 (0.6)	0 (0)	3 (1.3)	
AZT + 3TC + NVP	168 (35.1)	73 (29.4)	95 (41.3)	
D4T + 3TC + EFV	41 (8.6)	22 (8.9)	19 (8.3)	
D4T + 3TC + NVP	30 (6.3)	14 (5.6)	16 (7.0)	
TDF + 3TC + EFV	89 (18.6)	55 (22.2)	34 (14.8)	
TDF + 3TC + LVP	12 (2.5)	5 (2.0)	7 (3.0)	
TDF + 3TC + NVP	3 (0.6)	1 (0.4)	2 (0.9)	
3TC + DTG	1 (0.2)	0 (0)	1 (0.4)	
BIC/FTC/TAF	1 (0.2)	1 (0.4)	0 (0)	
EVG/c/FTC/TAF	3 (0.6)	0 (0)	3 (1.3)	
HDL, mmol/L	0.8 ± 0.3	0.9 ± 0.3	0.7 ± 0.3	0.032
AST, U/L	30.2 (22.0, 46.8)	29.0 (21.0, 40.0)	33.6 (23.0, 52.0)	0.007
ALT, U/L	27.0 (19.0, 44.1)	24.1 (17.4, 40.8)	29.0 (21.6, 49.4)	0.002
GGT, U/L	45.3 (26.0, 87.0)	41.0 (24.0, 81.0)	58.7 (41.8, 98.4)	0.011

Treatment time group, Time from discovery of HIV positivity to initiation of treatment; OHL, Oral Hairy Leukoplakia; PJP, Pneumocystis Jirovecii Pneumonia; CMV, Cytomegalovirus; SMZ-TMP, Sulfamethoxazole-Trimethoprim; HDL, High Density Lipoprotein; AST, Aspartate Aminotransferase; ALT, Alanine Aminotransferase; GGT, γ-Glutamyl transpeptidase.

The study excluded covariates with more than 20% missing data and filled in data with less than 20% missing using the random forest approach. The analysis of differences in the data before and after filling (Supplementary Material Table 1) showed no statistically significant differences in any variable. Subsequently, the filled patient data were randomly divided into a training set and a test set in a ratio of 6:4. An analysis of differences was performed between the two datasets (Supplementary Material Table 2). The differences in each variable were not statistically significant, and the data were balanced and comparable.

### Predictor selection

**Figure 3:**
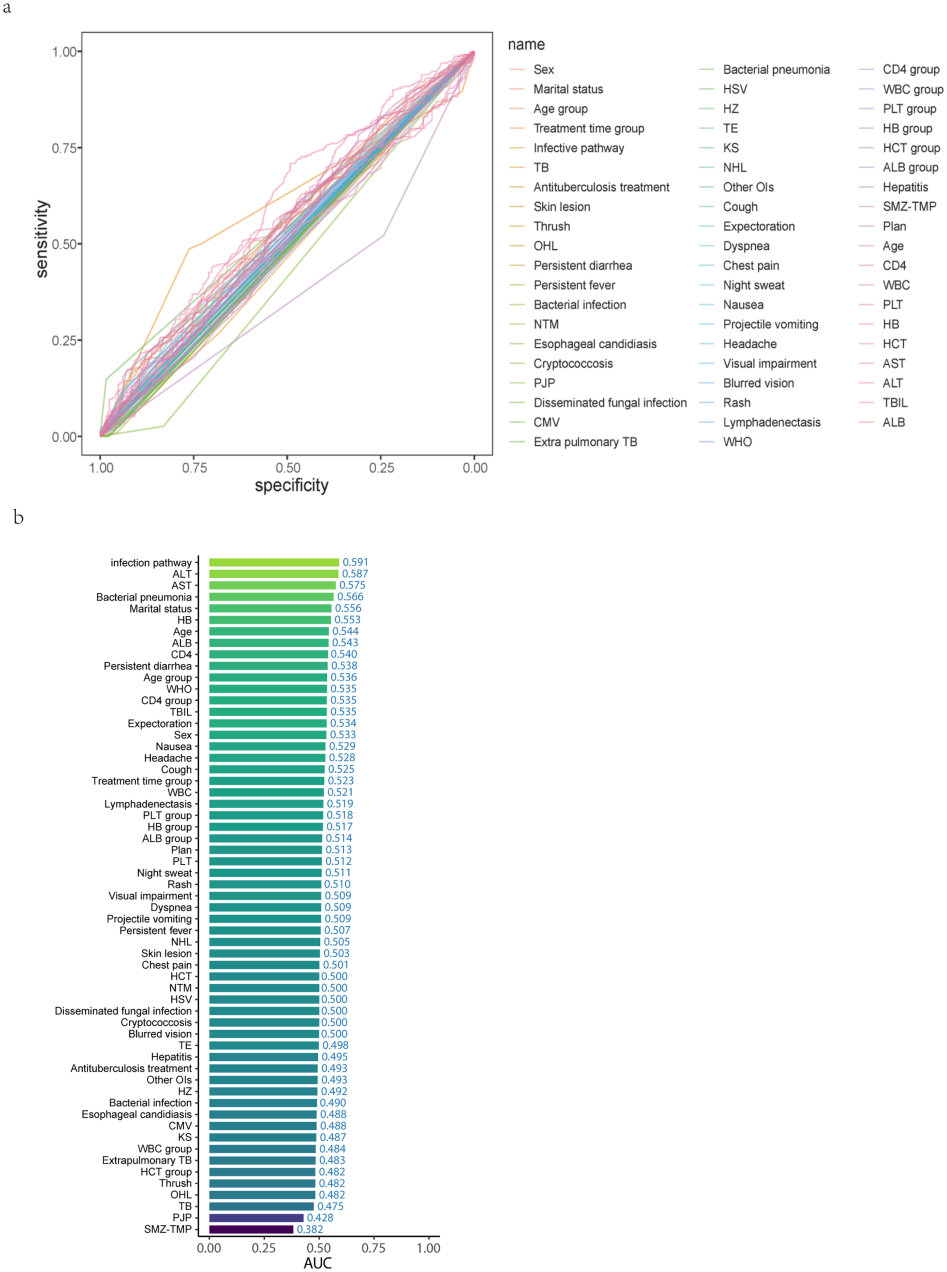
Initially determine the relationship between all independent variables and the ending variable and rank the contribution of the independent variables. **(a)** ROC curve for all independent variables. **(b)** AUC ranking of all independent variables. TB, Tuberculosis; OHL, Oral Hairy Leukoplakia; NTM, Nontuberculous Mycobacteria; PJP, Pneumocystis Jirovecii Pneumonia; CMV, Cytomegalovirus; HSV, Herpes Simplex Virus; HZ, Herpes Zoster; TE, Toxoplasmic Encephalitis; KS, Kaposi’s Sarcoma; NHL, Non-Hodgkin lymphoma; OIs, Opportunistic Infections; SMZ-TMP, Sulfamethoxazole-Trimethoprim; WBC, White Blood Cell; PLT, Platelet; HB, Hemoglobin; HCT, Hematocrit; AST, Aspartate Aminotransferase; ALT, Alanine Aminotransferase; TBIL, Total Bilirubin; ALB, Albumin.

First, an exploratory analysis of the data was conducted to plot ROC curves for all independent variables in the filled dataset (Figure 3a) to initially determine the relationships between all independent variables and the outcome variable. Then, the contributions of all independent variables were ranked (Figure 3b).

**Figure 4:**
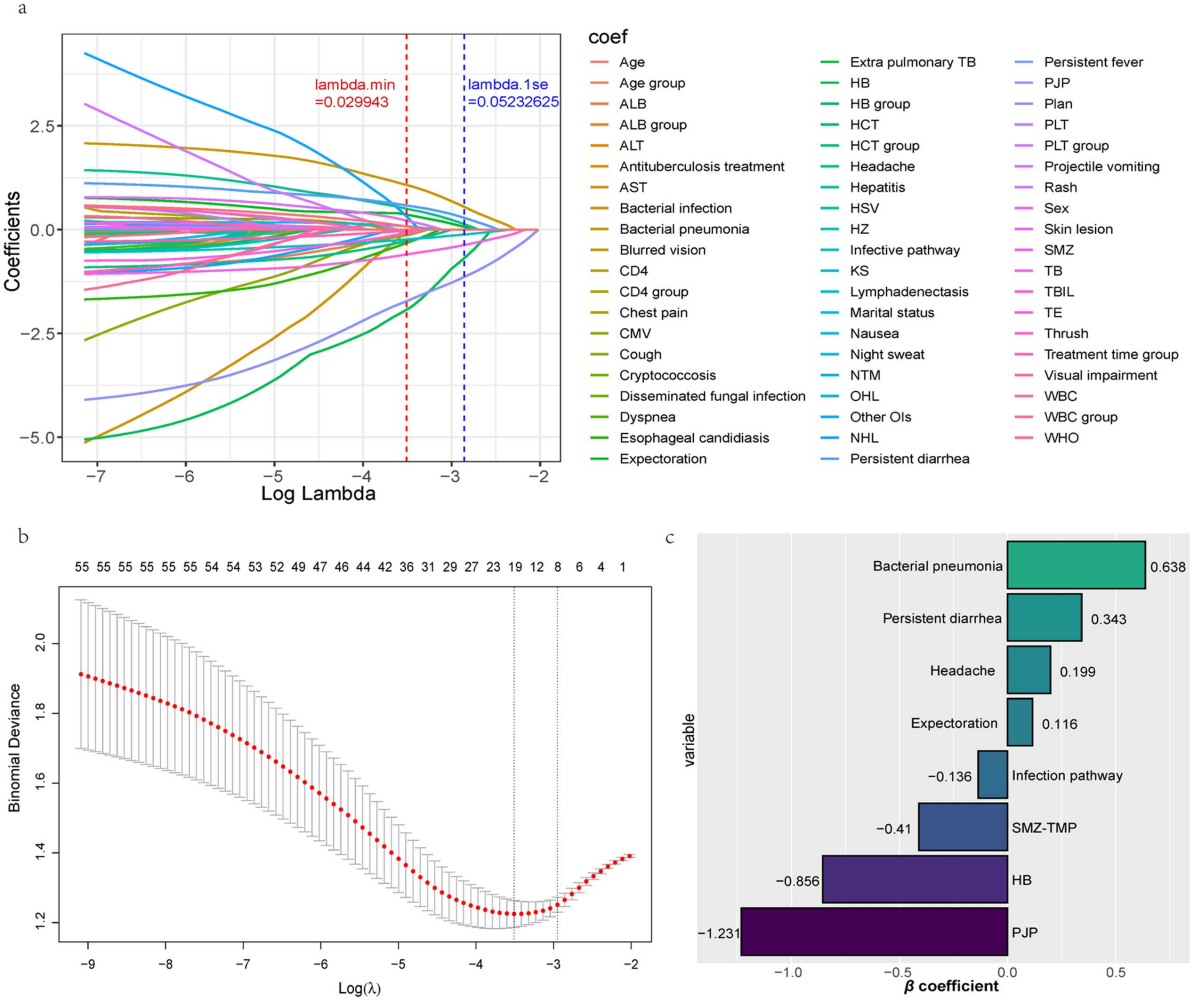
Screening of independent variables to obtain predictors. **(a)** Lambda plot. **(b)** Cross-validation plot. **(c)** Bar plots of positive and negative non-zero coefficients. ALB, Albumin; ALT, Alanine Aminotransferase; AST, Aspartate Aminotransferase; CMV, Cytomegalovirus; HB, Hemoglobin; HCT, Hematocrit; HSV, Herpes Simplex Virus; HZ, Herpes Zoster; KS, Kaposi’s Sarcoma; NTM, Nontuberculous Mycobacteria; OHL, Oral Hairy Leukoplakia; OIs, Opportunistic Infections; NHL, Non-Hodgkin lymphoma; PJP, Pneumocystis Jirovecii Pneumonia; PLT, Platelet; SMZ-TMP, Sulfamethoxazole-Trimethoprim; TB, Tuberculosis; TBIL, Total Bilirubin; TE, Toxoplasmic Encephalitis; WBC, White Blood Cell. Variable selection is based on the LASSO regression method using 10-fold cross-validation to determine the optimal penalty parameter λ. Variables screened are those with non-zero coefficients in the model. The horizontal axis is the penalty parameter λ, the vertical axis is the coefficients of the variables, and the vertical dashed line indicates the optimal value of λ selected through cross-validation.

Finally, lasso regression was performed on the training set to obtain the Lambda chart (Figure 4a) and the cross-validation diagram (Figure 4b). Predictors were screened from 59 independent variables, and non-zero coefficient positive and negative bar plots are shown in Figure 4c. Under the λ-1se dashed line, the model fit was good, and the number of predictors was appropriate. Ultimately, eight predictors were identified, including one continuous variable (HB) and seven categorical variables (bacterial pneumonia, persistent diarrhea, headache, expectoration, infection pathway, SMZ-TMP, PJP), and a min-max standardized transformation was performed for the continuous variable HB. Four variables were positively related to mortality (high-risk variables: bacterial pneumonia, persistent diarrhea, headache, expectoration), and four variables were negatively related to mortality (low-risk variables: infection pathway, SMZ-TMP, HB, PJP).

### Model building and evaluation

**Figure 5:**
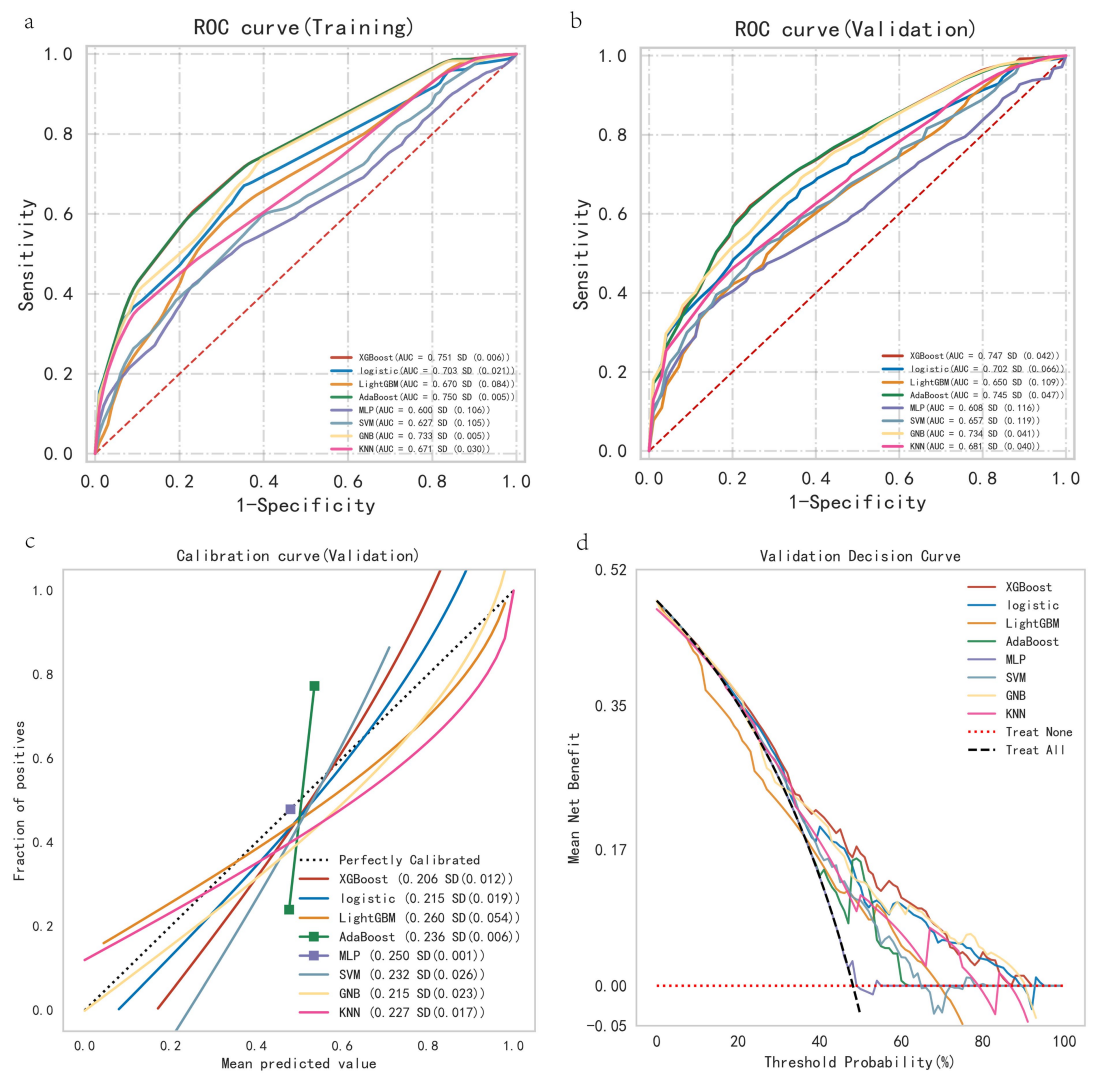
Performance evaluation of all models. **(a)** Training set ROC curve. **(b)** Ten-fold cross-validation ROC curve. **(c)** Ten-fold cross-validation calibration curve. **(d)** Ten-fold cross-validation DCA curve. Model XGBoost has the highest area under the ROC curve AUC and the most superior performance of the model. XGBoost has the lowest Brier score, which indicates that the closer the prediction probability is to the true outcome, the more accurate the model is. Model XGBoost has the highest clinical benefit for the DCA curve, with an interval of 20–90%.

This study selected seven machine learning algorithms (XGBoost, LightGBM, AdaBoost, MLP, SVM, GNB, KNN) and one traditional regression method (Logistic Regression) to build a patient mortality risk prediction model. The ROC curve, calibration curve, and DCA curve of the training set and ten-fold cross-validation were drawn (Figures 5a–d). According to Figure 5 and the cross-validation model parameter results (Table 2), it was found that among the eight models, the XGBoost model had the highest AUC, the lowest Brier score, the best-combined model parameter results, and the highest clinical benefit, so XGBoost was chosen for modeling. In order to optimize the performance of the XGBoost model, this study uses a grid search method for hyper-parameter tuning, with a nrounds of 200, max_depth of 3, eta of 0.01, gamma of 0.1, colsample_bytree of 0.7, min_child_weight of 3, and subsample of 0.7.

**Table 2:** Ten-fold cross-validation model performance parameters.

Model	AUC (SD)	Accuracy (SD)	Sensitivity (SD)	Specificity (SD)	F1 Score (SD)
XGBoost	0.747 (0.042)	0.680 (0.049)	0.674 (0.102)	0.734 (0.091)	0.685 (0.067)
Logistic	0.702 (0.066)	0.636 (0.071)	0.574 (0.152)	0.786 (0.130)	0.599 (0.119)
LightGBM	0.650 (0.109)	0.610 (0.106)	0.648 (0.201)	0.656 (0.286)	0.621 (0.080)
AdaBoost	0.745 (0.047)	0.665 (0.056)	0.670 (0.107)	0.734 (0.091)	0.704 (0.071)
MLP	0.608 (0.116)	0.594 (0.069)	0.470 (0.269)	0.807 (0.233)	0.494 (0.200)
SVM	0.657 (0.119)	0.636 (0.081)	0.735 (0.141)	0.604 (0.242)	0.659 (0.063)
GNB	0.734 (0.041)	0.663 (0.047)	0.635 (0.144)	0.770 (0.129)	0.639 (0.098)
KNN	0.681 (0.040)	0.590 (0.072)	0.535 (0.218)	0.768 (0.229)	NaN (NaN)

The XGBoost model has the best overall performance.

**Figure 6:**
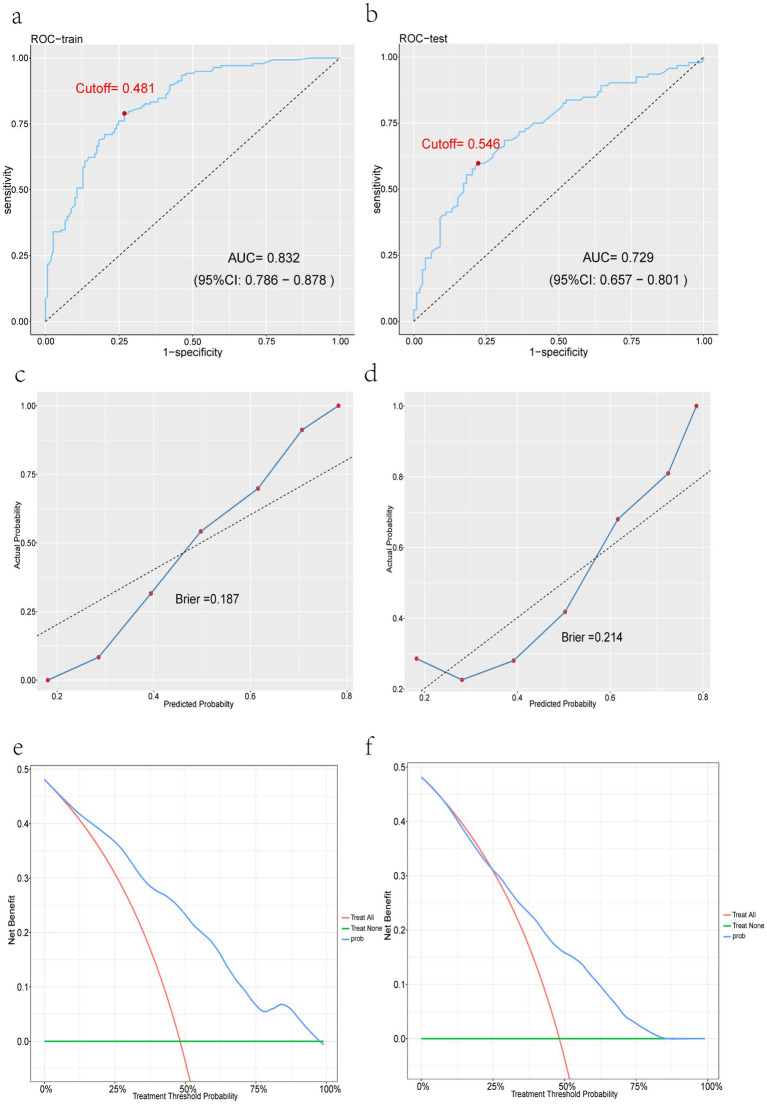
Performance evaluation of the XGBoost model. **(a)** XGBoost training set ROC curve. **(b)** XGBoost test set ROC curve. **(c)** XGBoost training set calibration curve. **(d)** XGBoost test set calibration curve. **(e)** XGBoost training set DCA curve. **(f)** XGBoost test set DCA curve.

**Figure 7:**
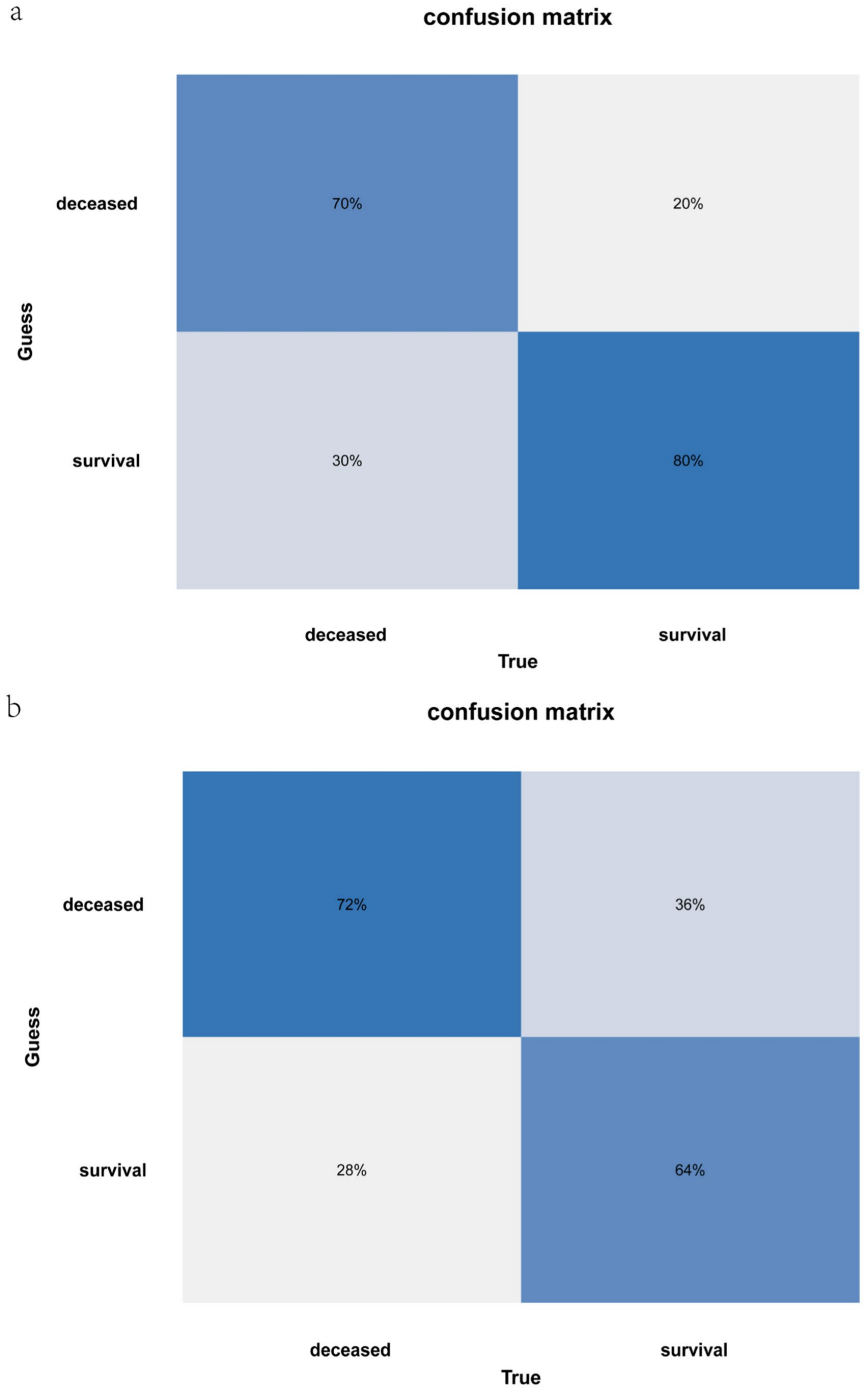
Accuracy and discriminative power of XGBoost model predictions. **(a)** Training set confusion matrix. **(b)** Test set confusion matrix. Confusion matrix plots show 70% and 72% sensitivity, reflecting the proportion of all individuals who were actually dead that the model model correctly predicted as dead. 80% and 64% specificity, referring to the proportion of all individuals who were actually alive that the model correctly predicted as alive.

Subsequently, the ROC curves and AUC values (Figures 6a,b), calibration curves and Brier scores (Figures 6c,d), and DCA curves (Figures 6e,f) of the training and test sets were plotted. Figures 6a,b shows that the training set AUC = 0.832, the test set AUC = 0.729, and the performance of the model is good; Figures 6c,d shows that the training set Brier = 0.187 and the test set Brier = 0.214 have low values and good fit; In Figures 6e,f, the blue lines indicate the clinical intervention benefits, and most of the blue lines are above the two thresholds, showing that the clinical benefits are relatively high. The accuracy and discriminant power of the model predictions were visualized using the confusion matrix plots (Figures 7a,b), and the performance of the prediction model was calculated (Table 3), including accuracy, sensitivity, specificity, positive predictive value, negative predictive value, and F1 score.

**Table 3:** Confusion matrix values.

	Accuracy	Sensitivity	Specificity	Pos pred value	Neg pred value	F1 score
Training set	0.753	0.703	0.799	0.744	0.764	0.732
Test set	0.675	0.717	0.636	0.708	0.647	0.680

**Figure 8:**
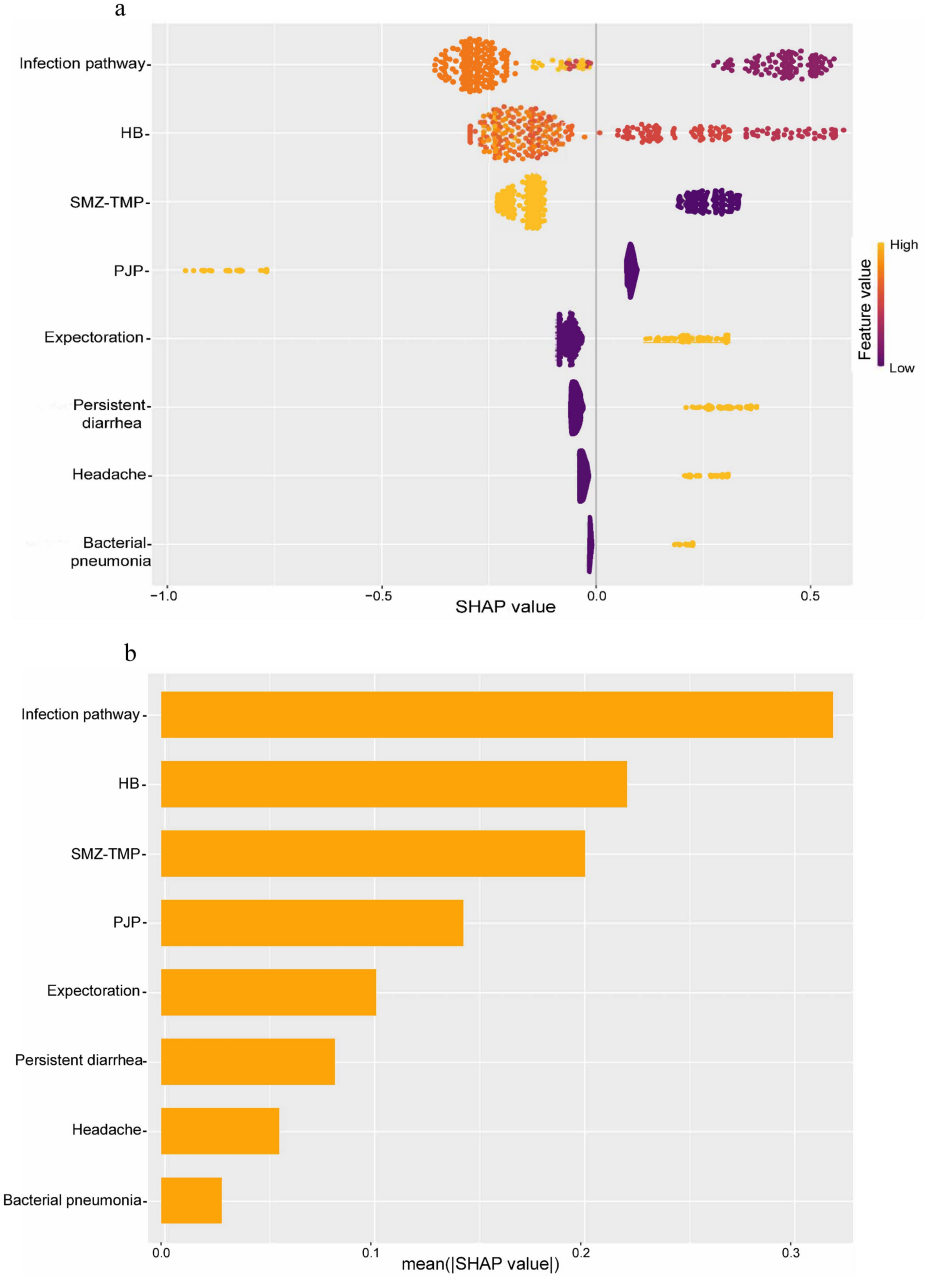
Importance and degree of contribution of model features. **(a)** SHAP beeswarm plot. **(b)** SHAP summary plot. HB, Hemoglobin; SMZ-TMP, Sulfamethoxazole-Trimethoprim; PJP, Pneumocystis Jirovecii Pneumonia. The horizontal axis of the SHAP beeswarm plot is the SHAP value, indicating the size and direction of the contribution of each feature to the prediction results, the color indicates the size of the feature value, the red dot feature value is large, the direction of its concentration, indicating the direction of the contribution to the prediction of the deaths. SHAP summary plot is to rank the importance of the variables, the importance of which decreases from the top to the bottom, and the longer the horizontal axis is, the longer the features are, indicating that they have a greater impact on the overall prediction of the model. The longer the horizontal axis and the longer the feature, indicating the greater its impact on the model's overall prediction.

**Figure 9:**
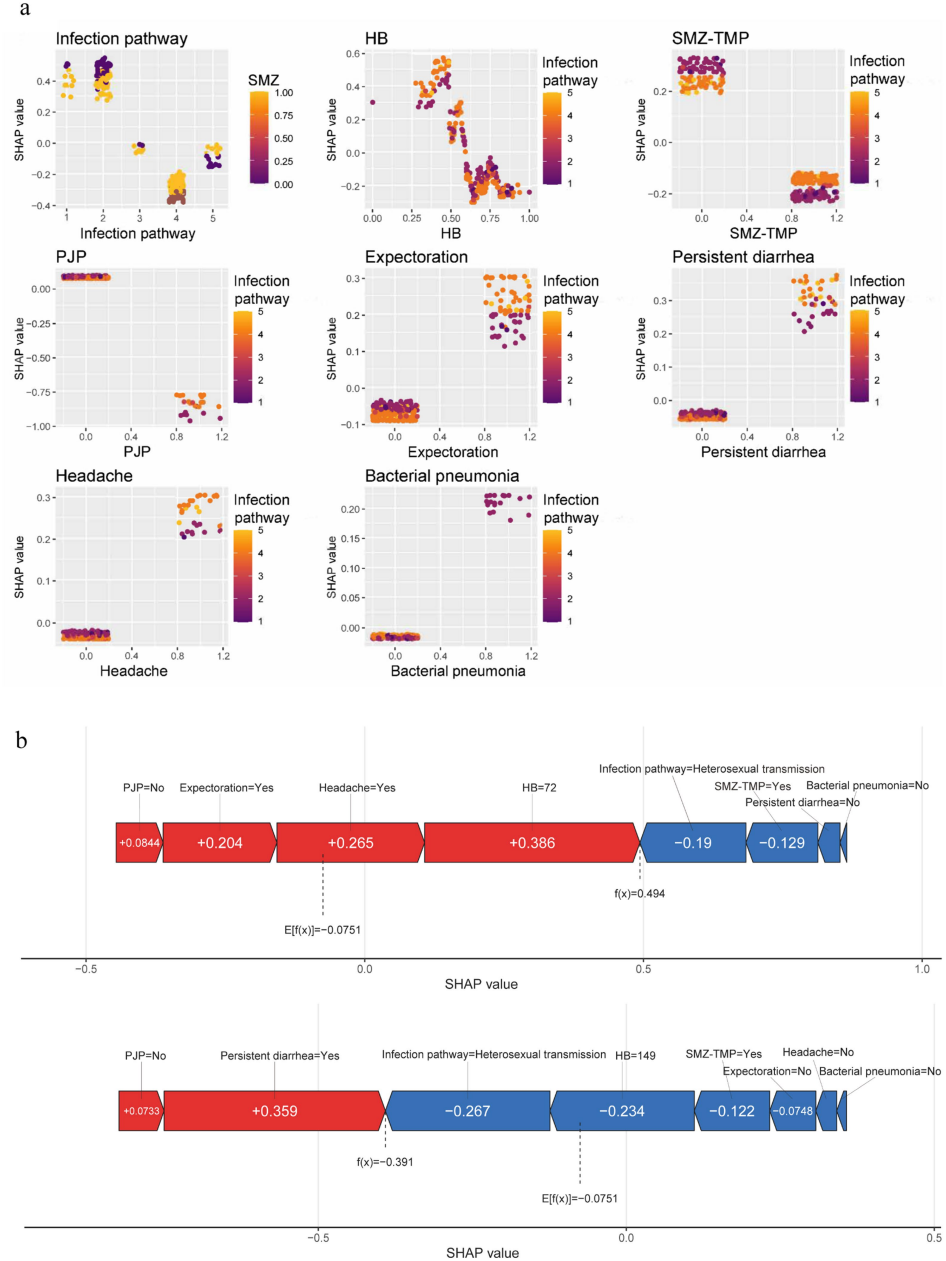
Interaction effects between features and model interpretation. **(a)** SHAP dependence plots. **(b)** SHAP force plot for Patient #10 & Patient #45. HB, Hemoglobin; SMZ-TMP, Sulfamethoxazole-Trimethoprim; PJP, Pneumocystis Jirovecii Pneumonia. The SHAP dependence plots show the interactions between variables, where the horizontal axis is the original value of a feature, the vertical axis is the corresponding SHAP value, and the color of the dot indicates the magnitude of the value of the other feature that is interacting. Red bars of the SHAP force plot indicate features that increase the predicted probability of death, and blue bars indicate features that decrease the predicted probability of death. The final SHAP values for each predictor are summed to score f(x), which is compared to the model's baseline prediction or expectation, E[f(x)]. f(x) greater than E[f(x)] indicates that the combination of these predictors results in a prediction that favors death when compared to the baseline prediction, and conversely the prediction favors survival.

The model was interpreted using the SHAP tool. For each sample, the model generated a predicted value. The SHAP value reflects the impact of each feature, indicating positive or negative effects. We plotted a SHAP beeswarm plot (Figure 8a) to visualize variable importance. The horizontal axis is the SHAP value, which indicates the magnitude and direction of each feature’s contribution to the prediction results; the color indicates the magnitude of the feature’s value (red for high values and blue for low values), with red dots concentrating in the positive direction and blue dots concentrating in the negative direction, which indicates that the higher the value of the feature, the higher the positive contribution it will make to the deaths. The SHAP summary plot (Figure 8b) was plotted to rank the importance of the variables, with importance decreasing from top to bottom, and the longer the horizontal axis, the longer the feature, indicating that it has a greater overall impact on the model’s predictions. A SHAP dependence plot (Figure 9a) was drawn to show the interaction between the variables, where the horizontal axis is the original value of a feature, the vertical axis is the corresponding SHAP value, and the color of the dots indicates the magnitude of the value of the other interacting feature. It can be seen from Figure 8b that infection pathway, HB, SMZ-TMP, and PJP are the top four important characteristics in terms of contribution, and they are negatively correlated with death. A higher value is associated with a lower risk of mortality; conversely, characteristics such as bacterial pneumonia, persistent diarrhea, headache, and expectoration were positively related to death, with higher values indicating a higher risk of death. Finally, we selected representative patients #10 and #45 to draw SHAP plots (Figure 9b). The prediction of death risk for patient #10 can be described by these characteristics: no PJP (SHAP value +0.0844), indicating a positive impact on the prediction of death. Expectoration symptoms (SHAP value +0.204) increased the likelihood of death. Headache symptoms (SHAP value +0.265) also increased the possibility of death. An HB level of 72 (SHAP value +0.386) was the most influential feature, significantly increasing the possibility of death. Heterosexual transmission (SHAP value −0.19) as the infection pathway reduced the possibility of death. Using SMZ-TMP (SHAP value −0.129) also reduced the likelihood of death. The absence of persistent diarrhea symptoms and bacterial pneumonia reduced the probability of death, but due to their small contribution, specific SHAP values were not shown in the figure. The final prediction score f(x) was 0.494, while the model’s baseline prediction or expectation E[f(x)] was −0.0751. This indicates that the combination of these characteristics resulted in a prediction leaning toward death compared to the baseline prediction. Red bars indicated features that increased the probability of predicted death, while blue bars indicated features that decreased it. The predictive description of the risk of death for patient #45 was similar to that described above, and the predictive results tended to be survival.

### External validation

**Figure 10:**
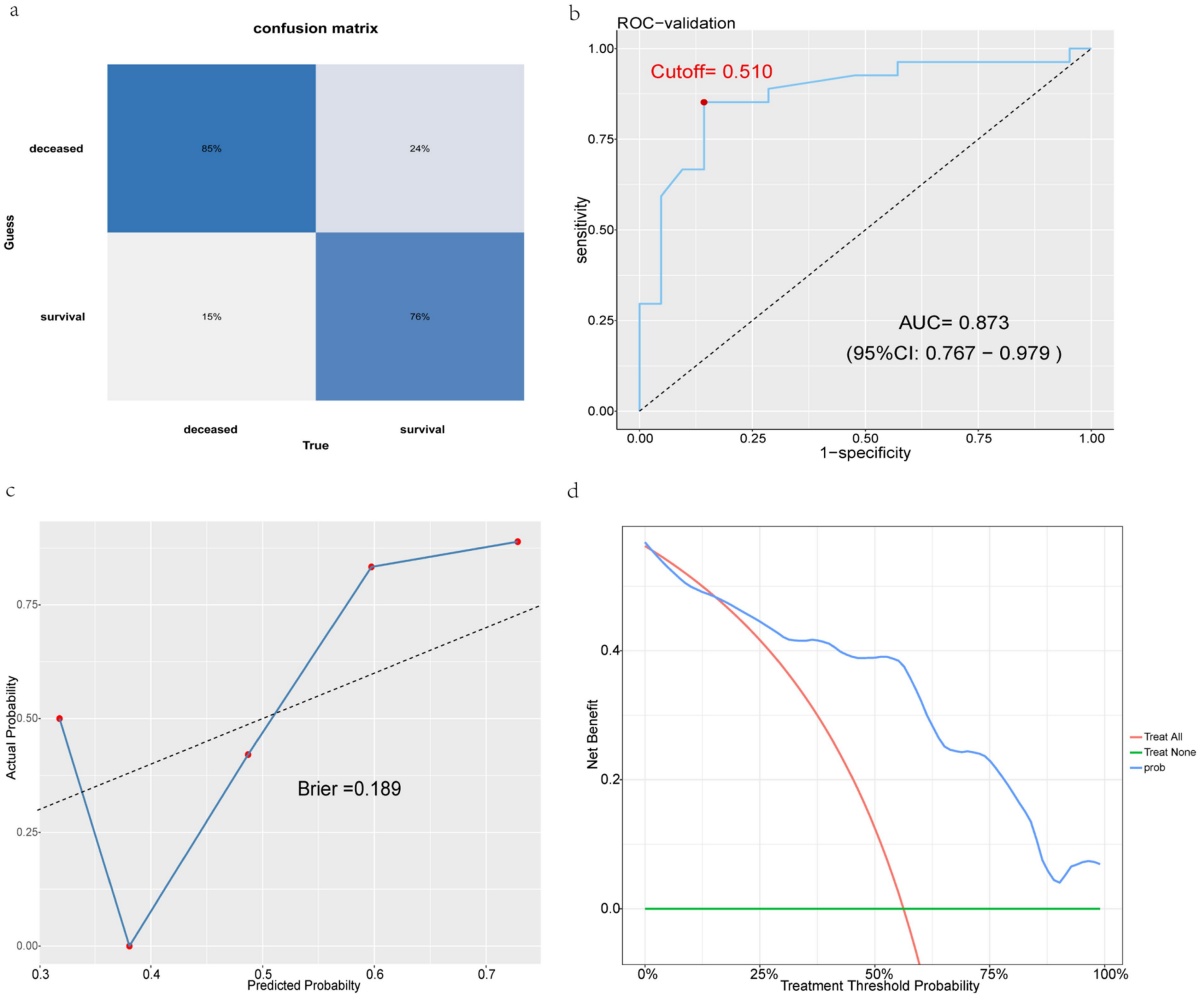
External validation shows excellent performance in models. **(a)** Validation set confusion matrix. **(b)** XGBoost validation set ROC curve. **(c)** XGBoost validation set calibration curve. **(d)** XGBoost validation set DCA curve.

In this study, we developed and internally validated a mortality risk prediction model based on the XGBoost algorithm. To further verify the generalization capability and practical application value of the model, we used the dataset of an external hospital for external validation. Forty-eight AIDS patients presenting with AIDS-related diseases or symptoms upon their first admission to the Shayibak District Branch of Urumqi Friendship Hospital (21 survivors and 27 deceased) were included in the external validation cohort. In external validation, the model also demonstrated superior predictive performance compared to traditional prediction methods. Specifically, it included a confusion matrix plot (Figure 10a), an ROC curve (Figure 10b), a calibration curve (Figure 10c), and a DCA curve (Figure 10d). Among them, important indicators such as accuracy, sensitivity, specificity, positive predictive value, negative predictive value, and F1 score were used to evaluate the performance of the model (Table 4).

**Table 4:** Validation confusion matrix values.

	Accuracy	Sensitivity	Specificity	Pos pred value	Neg pred value	F1 score
Validation set	0.813	0.852	0.762	0.800	0.821	0.836

## Discussion

In this study, the XGBoost algorithm demonstrated better discrimination (AUC = 0.751) compared to the other seven models (Logistic Regression, LightGBM, AdaBoost, etc.). After adjusting the parameters, the overall efficacy of the XGBoost model in predicting mortality risk was relatively high, reflected in the model’s ability to identify high-risk individuals. Specific indicators include prediction accuracy (Accuracy = 0.753), sensitivity (Sensitivity = 0.703), specificity (Specificity = 0.799), positive predictive value (Pos Pred Value = 0.744), negative predictive value (Neg Pred Value = 0.764), and F1 Score (F1 Score = 0.690). The AUC metric is used to measure the overall performance of the classification model, and the closer the value is to 1, the better the discriminative ability of the model. Meanwhile, since the goal of the study is to minimize the leakage of high-risk patients, Sensitivity and Negative Predictive Value are key indicators, and higher values indicate higher predictive reliability of the model. In addition, F1 Score serves as a balance between Positive Predictive Value/Precision and Sensitivity/Recall, with higher values representing a better measure of the model’s classification ability.

In subsequent external validation, we found that the performance on the external dataset was also relatively good, indicating that the model has good generalizability. The results of this study, although based on a region-specific sample, are considered to have some generalizability, especially among other groups of AIDS patients in similar regions. Additionally, the DCA curve of the model indicates that, compared to the intervention of all patients and the non-intervention of patients, the predictive model of the internally verified test set has higher clinical intervention benefits in predicting the risk of death for patients within the range of 25–85%. The predictive model of the external validation set has higher clinical intervention benefits in predicting the risk of death for patients within the range of 15–100%.

During the modeling process, it was observed that a reduction in HB increases the risk of death in patients. The potential reasons for this are as follows: HIV itself has myelosuppressive manifestations that lead to a decrease in HB, so a decrease in HB is one of the common clinical manifestations of HIV infection (40). In HIV patients, anemia is a factor in accelerated disease progression and reduced quality of life, and prolonged anemia also increases the risk of death (22, 41–43). Some studies have indicated that intravenous drug users are at a higher risk of death due to the route of infection, and bacterial pneumonia is the third most common cause of AIDS-related death, which is consistent with our findings (19, 44, 45). The present study found that expectoration, headache, and persistent diarrhea were associated with an increased risk of death. It is postulated that this may be due to the presence of expectoration symptoms suggestive of lung diseases, such as pneumonia and PJP; headache symptoms indicative of central nervous system disease; and persistent diarrhea symptoms commonly observed in patients with advanced AIDS. A study found that a decrease in HB may lead to a worse prognosis in patients with comorbid PJP, and anemia should be managed aggressively in AIDS patients with comorbid PJP if their HB is less than 90 g/L (46). The present study found that the prophylactic use of SMZ-TMP reduces the risk of death in patients with AIDS. This is because SMZ-TMP is effective in reducing the incidence of PJP, which is an important mortality factor for AIDS patients (47–49). Additionally, PJP was found to be a unique predictor showing a negative correlation in mortality risk prediction. The presumed reason is that PJP is a serious opportunistic infection and patients usually receive standardized treatment immediately upon diagnosis. This early diagnosis and intervention may significantly improve patient prognosis (50).

Some limitations of our study need to be acknowledged. First, the sample size of the study is relatively small, particularly for external validation. Although the patient data originate from two large hospitals, the sample size and number of outcome events may still limit the accuracy of extrapolating the results to other regions and may not be fully representative of all patient groups (e.g., different ages, genders, regions, etc.). Moreover, the validation cohort is derived from data from hospitals in a specific region, which may be subject to regional bias, disease distribution, and treatment differences. Patient characteristics (e.g., disease spectrum, treatments, lifestyle habits, etc.) in different regions may affect the predictive effectiveness of the model. Therefore, more external test sets from different hospitals or regions are needed to enhance the model’s robustness. As this study utilized retrospective data from hospital records, selection bias may have been introduced. Patients included in this study might represent those with more severe conditions or better treatment adherence, potentially limiting the generalizability of the findings to a broader population of AIDS patients. Second, the duration of follow-up from admission to the study cut-off point varied for each patient, which cannot be completely avoided in clinical practice, and which may have influenced the study results, particularly among patients with shorter follow-up times who may not have reached the study endpoints. Fortunately, external validation demonstrated excellent performance, suggesting the model is suitable for predicting the risk of death in the study’s patients, and related studies have confirmed that the performance of machine learning prediction models is not affected by the duration of follow-up (51). However, the predictive performance of the model still does not fully meet the expected level, which may be due to the limited sample size. Finally, different patients may require different treatment regimens, which may be adjusted during follow-up depending on the patient’s specific situation. Due to ethical considerations and the observational nature of the study, we could not ensure the impact of patient treatment regimens on outcomes.

The availability of high-quality, consistent patient data remains a major barrier to implementing predictive models in resource-limited settings. Missing data and reliance on historical datasets may result in information bias, selection bias, and temporal bias. Missing data may result in incomplete or inaccurate information about the variables on which the model relies, thus affecting the veracity and reliability of the analyzed results. Removal of missing values may tend to retain patient populations with complete data, leading to an underestimation or overestimation of the broad applicability of study results. The time span of historical data may introduce changes in medical technology, standards of care, and patient characteristics, affecting the comparability of studies and the applicability of results. The lack of computational infrastructure may also hinder the integration of machine learning models into routine clinical practice. Most importantly, ethical issues, such as the potential for predictive models to exacerbate health inequalities, need to be carefully considered. The application of predictive modeling in clinical decision-making may involve issues of patient privacy and autonomy and needs to be implemented with due consideration of ethical implications.

In future research, we will first continue to increase the sample size based on the existing results, conduct joint studies with several hospitals to enhance the robustness and accuracy of the model, and continue to optimize the model. Second, the follow-up duration will be controlled to ensure that patients are followed for a consistent length of time. Third, we will integrate more data types, such as patient imaging data and genetic data, to improve model performance. Additionally, we will explore advanced techniques such as feature engineering and ensemble methods to further improve the prediction performance. Feature engineering includes constructing interactive features to combine variables to improve data representation, e.g., BMI = weight/height^2^. Ensemble methods include combining XGBoost, LGBM, or neural networks to enhance model generalization. Fourth, we will explore the potential of applying the model to other related fields, such as predicting the risk of death in patients with other chronic diseases or using it in different epidemiological studies.

## Conclusion

In conclusion, the following variables were identified as important predictors of the risk of death in patients: infection pathway, HB, SMZ-TMP, PJP, expectoration, persistent diarrhea, headache, and bacterial pneumonia. The findings assist clinicians in assessing disease severity in various ways. This study may serve as a reference for future clinical studies and potential applications.

## Data Availability

The data analyzed in this study is subject to the following licenses/restrictions: due to relevant national laws and the specificity of the population involved in the study, the datasets used to generate the graphs and analyses in this study are not publicly available to protect the privacy of people living with HIV, but can be obtained from the corresponding authors upon reasonable request. Requests to access these datasets should be directed to Yiwei Chen, chen1973492893@126.com.
