# Supplementary material for: Machine learning-based prediction of mortality risk in AIDS patients with comorbid common AIDS-related diseases or symptoms
Source: Front Public Health. 2025 Mar 12;13:1544351. doi: 10.3389/fpubh.2025.1544351 (PMC11936937; doi:10.3389/fpubh.2025.1544351)
Supplement: Supplementary file 1 [file Table_1.docx]

**Supplementary Material Table 1 Differential analysis of data before and after filling**

| Variables | Before (n = 478) | After (n = 478) | statistic | *P* |
| --- | --- | --- | --- | --- |
| Marital status, n (%) |  |  | 0.002 | 0.999 |
| Single | 58 (12.2) | 58 (12.1) |  |  |
| Married or cohabiting | 338 (71.0) | 340 (71.1) |  |  |
| Divorced or widowed | 80 (16.8) | 80 (16.7) |  |  |
| CD4 group, n (%) |  |  | 0.022 | 0.999 |
| 1-99 | 223 (47.0) | 223 (46.7) |  |  |
| 100-199 | 119 (25.1) | 121 (25.3) |  |  |
| 200-349 | 91 (19.2) | 93 (19.5) |  |  |
| >350 | 41 (8.6) | 41 (8.6) |  |  |
| PLT group, n (%) |  |  | 0.008 | 0.928 |
| Normal | 398 (85.8) | 412 (86.2) |  |  |
| Abnormal | 66 (14.2) | 66 (13.8) |  |  |
| HCT group, n (%) |  |  | 0.025 | 0.874 |
| Normal | 119 (31.1) | 145 (30.3) |  |  |
| Abnormal | 264 (68.9) | 333 (69.7) |  |  |
| Hepatitis, n (%) |  |  | 0.298 | 0.585 |
| No | 301 (78.6) | 384 (80.3) |  |  |
| Yes | 82 (21.4) | 94 (19.7) |  |  |
| SMZ-TMP, n (%) |  |  | 0 | 0.996 |
| No | 170 (35.8) | 170 (35.6) |  |  |
| Yes | 305 (64.2) | 308 (64.4) |  |  |
| CD4, cells/μL | 111.5 (43.0, 214.0) | 113.0 (43, 215.5) | 112995 | 0.945 |
| WBC, 10^9^/L | 4.4 (3.2, 5.8) | 4.4 (3.2, 5.8) | 111479.5 | 0.934 |
| PLT, 10^9^/L | 175.0 (125.0, 228.5) | 175.0 (129.0, 228.0) | 110558.5 | 0.936 |
| HB, g/L | 117.0 (96.0, 133.0) | 116.0 (96.0, 133.0) | 112065 | 0.780 |
| HCT, % | 36.8 (31.0, 41.3) | 36.9 (31.0, 41.9) | 90877.5 | 0.856 |
| AST, U/L | 30.2 (22.0, 46.8) | 30.3 (22.0, 46.4) | 106420 | 0.987 |
| ALT, U/L | 27.0 (19.0, 44.1) | 27.0 (19.0, 44.0) | 107023 | 0.916 |
| TBIL, μmol/L | 9.6 (7.3, 13.2) | 9.7 (7.3, 13.2) | 104692.5 | 0.907 |
| ALB, g/L | 35.9 (31.0, 41.0) | 35.9 (31.0, 41.5) | 90800 | 0.839 |

Abbreviations: SMZ-TMP, Sulfamethoxazole-Trimethoprim; WBC, White Blood Cell; PLT, Platelet; HB, Hemoglobin; HCT, Hematocrit; AST, Aspartate Aminotransferase; ALT, Alanine Aminotransferase; TBIL, Total Bilirubin; ALB, Albumin.
