# Supplementary material for: Machine learning-based prediction of mortality risk in AIDS patients with comorbid common AIDS-related diseases or symptoms
Source: Front Public Health. 2025 Mar 12;13:1544351. doi: 10.3389/fpubh.2025.1544351 (PMC11936937; doi:10.3389/fpubh.2025.1544351)
Supplement: Supplementary file 2 [file Table_2.docx]

**Supplementary Material Table 2 Differential analysis between training and test sets**

| Variables | Total (n = 478) | Test (n = 191) | Training (n = 287) | statistic | *P* |
| --- | --- | --- | --- | --- | --- |
| Sex, n (%) |  |  |  | 0.895 | 0.344 |
| Female | 143 (29.9) | 52 (27.2) | 91 (31.7) |  |  |
| Male | 335 (70.1) | 139 (72.8) | 196 (68.3) |  |  |
| Marital status, n (%) |  |  |  | 0.556 | 0.757 |
| Single | 58 (12.1) | 21 (11.0) | 37 (12.9) |  |  |
| Married or cohabiting | 340 (71.1) | 136 (71.2) | 204 (71.1) |  |  |
| Divorced or widowed | 80 (16.7) | 34 (17.8) | 46 (16.0) |  |  |
| Age group, n (%) |  |  |  | 1.557 | 0.669 |
| 1-29 | 65 (13.6) | 24 (12.6) | 41 (14.3) |  |  |
| 30-39 | 200 (41.8) | 82 (42.9) | 118 (41.1) |  |  |
| 40-49 | 125 (26.2) | 46 (24.1) | 79 (27.5) |  |  |
| >50 | 88 (18.4) | 39 (20.4) | 49 (17.1) |  |  |
| Treatment time group, n (%) |  |  |  | 1.757 | 0.624 |
| 0-30 days | 238 (49.8) | 96 (50.3) | 142 (49.5) |  |  |
| 31-90 days | 108 (22.6) | 47 (24.6) | 61 (21.3) |  |  |
| 91-365 days | 69 (14.4) | 27 (14.1) | 42 (14.6) |  |  |
| >365 days | 63 (13.2) | 21 (11.0) | 42 (14.6) |  |  |
| Infection pathway, n (%) |  |  |  | Fisher | 0.727 |
| Blood-borne (transfusion + apheresis) | 22 (4.6) | 10 (5.2) | 12 (4.2) |  |  |
| Intravenous drug addiction | 149 (31.2) | 55 (28.8) | 94 (32.8) |  |  |
| homosexual transmission | 11 (2.3) | 3 (1.6) | 8 (2.8) |  |  |
| Heterosexual transmission | 264 (55.2) | 111 (58.1) | 153 (53.3) |  |  |
| Other (mother-to-child transmission + | 32 (6.7) | 12 (6.3) | 20 (7.0) |  |  |
| unknown route) |  |  |  |  |  |
| TB, n (%) |  |  |  | 0.162 | 0.687 |
| No | 339 (70.9) | 133 (69.6) | 206 (71.8) |  |  |
| Yes | 139 (29.1) | 58 (30.4) | 81 (28.2) |  |  |
| Antituberculosis treatment, n (%) |  |  |  | 0.5 | 0.479 |
| No | 350 (73.2) | 136 (71.2) | 214 (74.6) |  |  |
| Yes | 128 (26.8) | 55 (28.8) | 73 (25.4) |  |  |
| Skin lesion, n (%) |  |  |  | 0.074 | 0.786 |
| No | 463 (96.9) | 184 (96.3) | 279 (97.2) |  |  |
| Yes | 15 (3.1) | 7 (3.7) | 8 (2.8) |  |  |
| Thrush, n (%) |  |  |  | 0.262 | 0.609 |
| No | 438 (91.6) | 173 (90.6) | 265 (92.3) |  |  |
| Yes | 40 (8.4) | 18 (9.4) | 22 (7.7) |  |  |
| OHL, n (%) |  |  |  | 3.601 | 0.058 |
| No | 465 (97.3) | 182 (95.3) | 283 (98.6) |  |  |
| Yes | 13 (2.7) | 9 (4.7) | 4 (1.4) |  |  |
| Bacterial infection, n (%) |  |  |  | 0.173 | 0.393 |
| No | 473 (99.0) | 188 (98.4) | 285 (99.3) |  |  |
| Yes | 5 (1.0) | 3 (1.6) | 2 (0.7) |  |  |
| Esophageal candidiasis, n (%) |  |  |  | 1.462 | 0.486 |
| No | 470 (98.3) | 189 (99.0) | 281 (97.9) |  |  |
| Yes | 8 (1.7) | 2 (1.0) | 6 (2.1) |  |  |
| PJP, n (%) |  |  |  | 1.802 | 0.180 |
| No | 430 (90.0) | 167 (87.4) | 263 (91.6) |  |  |
| Yes | 48 (10.0) | 24 (12.6) | 24 (8.4) |  |  |
| Extrapulmonary TB, n (%) |  |  |  | 0.001 | 0.971 |
| No | 422 (88.3) | 168 (88.0) | 254 (88.5) |  |  |
| Yes | 56 (11.7) | 23 (12.0) | 33 (11.5) |  |  |
| Bacterial pneumonia, n (%) |  |  |  | 2.220 | 0.136 |
| No | 440 (92.1) | 171 (89.5) | 269 (93.7) |  |  |
| Yes | 38 (7.9) | 20 (10.5) | 18 (6.3) |  |  |
| HZ, n (%) |  |  |  | 0.178 | 0.673 |
| No | 447 (93.5) | 177 (92.7) | 270 (94.1) |  |  |
| Yes | 31 (6.5) | 14 (7.3) | 17 (5.9) |  |  |
| KS, n (%) |  |  |  | 0.188 | 0.664 |
| No | 459 (96.0) | 182 (95.3) | 277 (96.5) |  |  |
| Yes | 19 (4.0) | 9 (4.7) | 10 (3.5) |  |  |
| NHL, n (%) |  |  |  | Fisher | 0.307 |
| No | 474 (99.2) | 188 (98.4) | 286 (99.7) |  |  |
| Yes | 4 (0.8) | 3 (1.6) | 1 (0.3) |  |  |
| Other OIs, n (%) |  |  |  | 0.05 | 0.824 |
| No | 435 (91.0) | 175 (91.6) | 260 (90.6) |  |  |
| Yes | 43 (9.0) | 16 (8.4) | 27 (9.4) |  |  |
| Persistent diarrhea, n (%) |  |  |  | 0.173 | 0.678 |
| No | 418 (87.4) | 169 (88.5) | 249 (86.8) |  |  |
| Yes | 60 (12.6) | 22 (11.5) | 38 (13.2) |  |  |
| Persistent fever, n (%) |  |  |  | 1.462 | 0.227 |
| No | 342 (71.5) | 143 (74.9) | 199 (69.3) |  |  |
| Yes | 136 (28.5) | 48 (25.1) | 88 (30.7) |  |  |
| Cough, n (%) |  |  |  | 0.048 | 0.826 |
| No | 349 (73.0) | 141 (73.8) | 208 (72.5) |  |  |
| Yes | 129 (27.0) | 50 (26.2) | 79 (27.5) |  |  |
| Expectoration, n (%) |  |  |  | 0.021 | 0.884 |
| No | 370 (77.4) | 149 (78.0) | 221 (77.0) |  |  |
| Yes | 108 (22.6) | 42 (22.0) | 66 (23.0) |  |  |
| Chest pain, n (%) |  |  |  | 0.359 | 0.549 |
| No | 441 (92.3) | 174 (91.1) | 267 (93.0) |  |  |
| Yes | 37 (7.7) | 17 (8.9) | 20 (7.0) |  |  |
| Night sweat, n (%) |  |  |  | 0.398 | 0.528 |
| No | 367 (76.8) | 150 (78.5) | 217 (75.6) |  |  |
| Yes | 111 (23.2) | 41 (21.5) | 70 (24.4) |  |  |
| Nausea, n (%) |  |  |  | 0.001 | 0.970 |
| No | 432 (90.4) | 172 (90.1) | 260 (90.6) |  |  |
| Yes | 46 (9.6) | 19 (9.9) | 27 (9.4) |  |  |
| Headache, n (%) |  |  |  | 3.850 | 0.050 |
| No | 440 (92.1) | 182 (95.3) | 258 (89.9) |  |  |
| Yes | 38 (7.9) | 9 (4.7) | 29 (10.1) |  |  |
| Visual impairment, n (%) |  |  |  | Fisher | 0.770 |
| No | 466 (97.5) | 187 (97.9) | 279 (97.2) |  |  |
| Yes | 12 (2.5) | 4 (2.1) | 8 (2.8) |  |  |
| Rash, n (%) |  |  |  | 0.649 | 0.421 |
| No | 433 (90.6) | 170 (89.0) | 263 (91.6) |  |  |
| Yes | 45 (9.4) | 21 (11.0) | 24 (8.4) |  |  |
| Lymphadenectasis, n (%) |  |  |  | 0.536 | 0.464 |
| No | 402 (84.1) | 164 (85.9) | 238 (82.9) |  |  |
| Yes | 76 (15.9) | 27 (14.1) | 49 (17.1) |  |  |
| WHO, n (%) |  |  |  | 0.657 | 0.883 |
| Stage 1 | 22 (4.6) | 8 (4.2) | 14 (4.9) |  |  |
| Stage 2 | 401 (83.9) | 163 (85.3) | 238 (82.9) |  |  |
| Stage 3 | 31 (6.5) | 12 (6.3) | 19 (6.6) |  |  |
| Stage 4 | 24 (5.0) | 8 (4.2) | 16 (5.6) |  |  |
| CD4 group, n (%) |  |  |  | 4.771 | 0.189 |
| 1-99 | 223 (46.7) | 86 (45.0) | 137 (47.7) |  |  |
| 100-199 | 121 (25.3) | 57 (29.8) | 64 (22.3) |  |  |
| 200-349 | 93 (19.5) | 36 (18.8) | 57 (19.9) |  |  |
| >350 | 41 (8.6) | 12 (6.3) | 29 (10.1) |  |  |
| WBC group, n (%) |  |  |  | 0.626 | 0.429 |
| Normal | 277 (57.9) | 106 (55.5) | 171 (59.6) |  |  |
| Abnormal | 201 (42.1) | 85 (44.5) | 116 (40.4) |  |  |
| PLT group, n (%) |  |  |  | 2.527 | 0.112 |
| Normal | 412 (86.2) | 171 (89.5) | 241 (84.0) |  |  |
| Abnormal | 66 (13.8) | 20 (10.5) | 46 (16.0) |  |  |
| HB group, n (%) |  |  |  | 0.51 | 0.475 |
| Normal | 135 (28.2) | 50 (26.2) | 85 (29.6) |  |  |
| Abnormal | 343 (71.8) | 141 (73.8) | 202 (70.4) |  |  |
| HCT group, n (%) |  |  |  | 0.085 | 0.770 |
| Normal | 145 (30.3) | 56 (29.3) | 89 (31.0) |  |  |
| Abnormal | 333 (69.7) | 135 (70.7) | 198 (69.0) |  |  |
| ALB group, n (%) |  |  |  | 0.383 | 0.536 |
| Normal | 144 (30.1) | 54 (28.3) | 90 (31.4) |  |  |
| Abnormal | 334 (69.9) | 137 (71.7) | 197 (68.6) |  |  |
| Hepatitis, n (%) |  |  |  | 0.517 | 0.472 |
| No | 384 (80.3) | 157 (82.2) | 227 (79.1) |  |  |
| Yes | 94 (19.7) | 34 (17.8) | 60 (20.9) |  |  |
| SMZ-TMP, n (%) |  |  |  | 1.121 | 0.290 |
| No | 170 (35.6) | 62 (32.5) | 108 (37.6) |  |  |
| Yes | 308 (64.4) | 129 (67.5) | 179 (62.4) |  |  |
| Plan, n (%) |  |  |  | Fisher | 0.687 |
| AZT+3TC+DDI | 1 (0.2) | 0 (0) | 1 (0.3) |  |  |
| AZT+3TC+EFV | 126 (26.4) | 49 (25.7) | 77 (26.8) |  |  |
| AZT+3TC+LVP | 3 (0.6) | 2 (1.0) | 1 (0.3) |  |  |
| AZT+3TC+NVP | 168 (35.1) | 65 (34.0) | 103 (35.9) |  |  |
| D4T+3TC+EFV | 41 (8.6) | 17 (8.9) | 24 (8.4) |  |  |
| D4T+3TC+NVP | 30 (6.3) | 11 (5.8) | 19 (6.6) |  |  |
| TDF+3TC+EFV | 89 (18.6) | 35 (18.3) | 54 (18.8) |  |  |
| TDF+3TC+LVP | 12 (2.5) | 8 (4.2) | 4 (1.4) |  |  |
| TDF+3TC+NVP | 3 (0.6) | 1 (0.5) | 2 (0.7) |  |  |
| 3TC+DTG | 1 (0.2) | 1 (0.5) | 0 (0) |  |  |
| BIC/FTC/TAF | 1 (0.2) | 0 (0) | 1 (0.3) |  |  |
| EVG/c/FTC/TAF | 3 (0.6) | 2 (1.0) | 1 (0.3) |  |  |
| Age, year | 38.0 (33.0, 47.0) | 38.0 (33.0, 47.0) | 38.0 (31.0, 46.0) | 28995 | 0.283 |
| CD4, cells/μL | 113.0 (43, 215.5) | 116.0 (45.0, 197.0) | 110.0 (41.0, 228.5) | 27673.5 | 0.858 |
| WBC, 10^9^/L | 4.4 (3.2, 5.8) | 4.3 (3.3, 5.8) | 4.4 (3.1, 5.8) | 27850 | 0.766 |
| PLT, 10^9^/L | 175.0 (129.0, 228.0) | 175.0 (132.5, 223.5) | 175.0 (121.0, 231.0) | 28297.5 | 0.548 |
| HB, g/L | 114.3 ± 25.6 | 114.0 ± 24.6 | 114.5 ± 26.2 | -0.22 | 0.826 |
| HCT, % | 36.9 (31.0, 41.9) | 35.4 (31.0, 41.0) | 37 (31.5, 42.0) | 25336.5 | 0.161 |
| AST, U/L | 30.3 (22.0, 46.4) | 31.0 (22.1, 49.3) | 30.0 (21.8, 44.7) | 28159.5 | 0.612 |
| ALT, U/L | 27.0 (19.0, 44.0) | 29.6 (19.8, 44.9) | 26.6 (18.0, 41.8) | 29469 | 0.164 |
| TBIL, μmol/L | 9.7 (7.3, 13.2) | 9.4 (7.2, 12.9) | 9.9 (7.6, 13.4) | 25101.5 | 0.119 |
| ALB, g/L | 35.9 (31.0, 41.5) | 35.7 (28.7, 40.3) | 36.3 (31.9, 41.8) | 24708 | 0.068 |

Abbreviations: Treatment time group, Time from discovery of HIV positivity to initiation of treatment; TB, Tuberculosis; OHL, Oral Hairy Leukoplakia; NTM, Nontuberculous Mycobacteria; PJP, Pneumocystis Jirovecii Pneumonia; CMV, Cytomegalovirus; HSV, Herpes Simplex Virus; HZ, Herpes Zoster; TE, Toxoplasmic Encephalitis; KS, Kaposi’s Sarcoma; NHL, Non-Hodgkin lymphoma; OIs, Opportunistic Infections; SMZ-TMP, Sulfamethoxazole-Trimethoprim; WBC, White Blood Cell; PLT, Platelet; HB, Hemoglobin; HCT, Hematocrit; AST, Aspartate Aminotransferase; ALT, Alanine Aminotransferase; TBIL, Total Bilirubin; ALB, Albumin;
